# Robust human genetic evidence supporting causal effects of FGF21 on reducing alcohol consuming behaviours

**DOI:** 10.1186/s12916-026-04807-x

**Published:** 2026-03-23

**Authors:** Héléne T. Cronjé, Sile Hu, Rachel Gurrell, Marijana Vujkovic, Susanna C. Larsson, Richard Mason, Richard Butt, Dipender Gill

**Affiliations:** 1 Sequoia Genetics, London, UK; 2 Apollo Therapeutics, Cambridge, UK; 3 Corporal Michael J. Crescenz VA Medical Center, Philadelphia, PA USA; 4 Department of Medicine, Division of Translational Medicine and Human Genetics, University of Pennsylvania Perelman School of Medicine, Philadelphia, PA USA; 5 Department of Epidemiology, University of Pennsylvania Perelman School of Medicine, Philadelphia, PA USA; 6 Unit of Cardiovascular and Nutritional Epidemiology, Institute of Environmental Medicine, Karolinska Institute, Stockholm, Sweden; 7 Medical Epidemiology, Department of Surgical Sciences, Uppsala University, Uppsala, Sweden; 8 Centre for Health Leadership & Enterprise, Cambridge Judge Business School, University of Cambridge, Cambridge, UK

**Keywords:** Alcohol use disorder, Fibroblast growth factor 21, Human genetics

## Abstract

**Background:**

Alcohol use disorder (AUD) represents a tremendous societal burden, yet few efficacious therapies are available and widely used. Pre-clinical and human observational data support fibroblast growth factor 21 (FGF21) as a promising therapeutic target for the treatment of AUD. The objective of this study is to identify a robust genetic instrument for FGF21 agonism and leverage it to explore the effects of FGF21 agonism on AUD and related traits, as well as metabolic outcomes more widely.

**Methods:**

We first compared associations with the positive control outcomes of liver fat and liver cirrhosis risk for the FGF21 cis-protein quantitative trait locus (cis-pQTL) (rs838131) to those for the common allele FGF21 L174P missense variant (rs739320). Having identified the L174P missense variant as a plausible genetic instrument, we subsequently performed association analyses investigating effects on AUD, related traits, and metabolic outcomes more widely. Finally, we performed colocalisation analyses to test whether observed association results reflect a causal mechanism that overlaps with the clinical effects of FGF21 on liver fat and liver cirrhosis.

**Results:**

Consistent association and colocalisation evidence support a protective association between genetically predicted FGF21 agonism and alcohol consumption (association *p* = 1 × 10^−18^, colocalisation posterior probability = 0.90), problematic alcohol use (association *p* = 0.02, posterior probability = 0.64), and AUD (association *p* = 9 × 10^−8^, posterior probability = 0.97). Similar evidence was also observed for favourable effects of FGF21 on improving kidney function, lowering triglyceride levels, lowering proportional energy intake from carbohydrates, increasing proportional energy intake from protein and fat, increasing body weight and lowering waist-to-hip ratio.

**Conclusions:**

This study identifies a genetic instrument for FGF21 effects to provide causal human evidence supporting favourable effects of FGF21 analogues for the treatment of AUD and related traits, as well as on metabolic outcomes more broadly. Further clinical study is duly warranted.

**Supplementary Information:**

The online version contains supplementary material available at 10.1186/s12916-026-04807-x.

## Background

Fibroblast growth factor 21 (FGF21) is a circulating hormone that is primarily produced in the liver and has been implicated in glucose and lipid metabolism [1]. Despite initially being pursued as a therapeutic target in diabetes and obesity with limited success [2], FGF21 analogues have demonstrated promising signs of efficacy for treating severe hypertriglyceridemia [3], metabolic dysfunction-associated steatohepatitis (MASH) and liver cirrhosis [4].

Given the pleiotropic effects of FGF21 [5], its analogues may also offer additional therapeutic opportunities. Specifically, pre-clinical studies have supported effects of FGF21 on broadly suppressing alcohol consumption behaviours through mechanisms that affect alcohol palatability and modulation of neuronal activity in the nucleus accumbens [6]. Such data have highlighted FGF21 analogues as a potential therapy for treating alcohol use disorder (AUD) [7]. With 7% of the global population aged over 15 years estimated to have lived with AUD [8], and few efficacious and safe treatments currently available [9], developing novel therapies remains a research and development priority.

The objective of this study is to leverage large-scale human genetic data to explore the causal evidence for effects of FGF21 on alcohol consumption behaviours and AUD, as well as related metabolic traits. We confirm the validity of our genetic instrument for FGF21 by demonstrating its associations with liver fat and liver cirrhosis as positive control analyses. Our findings generate important insights that support the clinical development of FGF21 analogues for the treatment of AUD.

## Methods

### Study overview

For the first step in this study, a robust genetic instrument for FGF21 agonism was identified by considering potential candidates and investigating their genetic association estimates with the positive control outcomes of liver fat and liver cirrhosis risk. FGF21 perturbation was subsequently instrumented using *FGF21*single-nucleotide polymorphism (SNP) rs739320. This missense variant results in a leucine to proline amino acid change in position 174, and has been shown to mimic the clinical effects of FGF21 analogues, including lowering liver fat percentage and cirrhosis risk [10–12]. This SNP is also the lead genome-wide significant signal at the *FGF21*gene in the current largest genome-wide association study (GWAS) of cirrhosis [11].

**Fig. 1:**
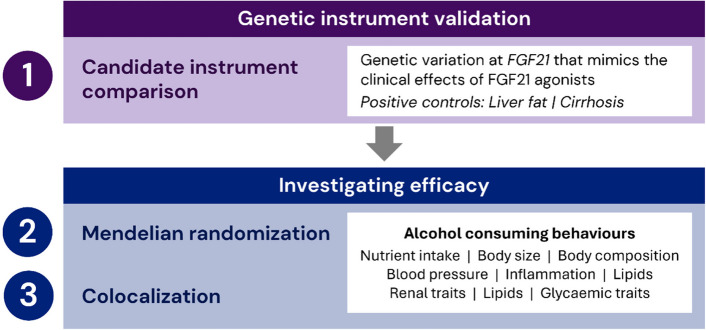
Study design

Primary outcomes of interest were alcohol consumption, problematic alcohol use (PAU) and AUD. Related behavioural, cardiometabolic and renal biomarkers and outcomes are investigated as secondary outcomes. As a sensitivity analysis, statistical colocalisation analyses were performed to evaluate whether genetic association evidence may be biased by confounding variants in linkage disequilibrium to rs739320. An overview of the study design is provided in Fig. 1.

### Data sources

Genetic association estimates for circulating FGF21 levels [13], magnetic resonance imaging (MRI)-derived liver fat percentage [10], cirrhosis liability [12], proportional macronutrient intake [14], alcohol consuming behaviour [15, 16], substance use [17], body size [18], body composition [19, 20], bone mineral density [21], blood pressure [22], biomarkers of inflammation [23, 24], renal function [25–28], chronic kidney disease risk [17], blood lipids [29] and glycaemic traits [30] were obtained from published studies.

All included studies had obtained the requisite ethical approval and participant consent for summary data distribution. For traits studied in our previous work [31], we leveraged updated (i.e. recent GWASs using data overlapping with those used before) or independent data where possible. Table 1 provides an overview of all data sources, including a citation to their original publications wherein additional information can be accessed.

**Table 1:** Data sources

**Outcome trait**	**Sample size**	**Cohort(s)**	**Citation**	**Notes**^†^
**Instrument generation**				
**FGF21**, NPX	54,219	UKB	[13]	Novel
**Liver fat**, %	32,858	UKB	[10]	
**Cirrhosis**	18,265/1,782,047	MA	[12]	Novel
** Nutrient intake**				
**Carbohydrate, fat, protein**, % TE	191,157	CHARGE, UKB	[14]	Updated
**Alcohol consuming behaviour and substance use**				
**Alcohol**, drinks per week	665,346	MA	[16]	Updated
**Problematic alcohol use**	165,952/762,876	MA	[15]	Novel
**Alcohol use disorder**	37,802/836,637	FG, UKB	[17]	Novel
**Smoking**, cigarettes per day	326,497	FG, UKB	[17]	Novel
**Smoking dependency**	23,706/887,736	FG, UKB	[17]	Novel
**SUD excl. alcohol**	8840/468,475	FG	[17]	Novel
**Body size**				
**Height**, cm	601,127	MVP	[18]	Independent
**Weight**, kg	602,768	MVP	[18]	Independent
**BMI**, kg/m^2^	601,007	MVP	[18]	Independent
**Body composition**				
**Total lean mass**, kg	413,158	UKB	[19]	Novel
**Waist-to-hip-ratio**	697,734	GIANT	[20]	
**Heel bone mineral density**, g/cm^2^	394,929	UKB	[21]	Novel
**Blood pressure**				
** Systolic BP, diastolic BP, pulse pressure**, mmHg	1,028,980	UKB, ICBP, MVP & BioVU	[22]	Updated
**Inflammation**				
** C-reactive protein**, log(mg/l)	575,531	CHARGE, UKB	[23]	Novel
** White blood cell count**, 10^9^ cells /L	746,667	BCC	[24]	Novel
**Renal traits**				
** eGFR**_**creat**_, ml/min/1.73^2^	1,508,659	MA	[26]	Updated
** eGFR**_**cys**-c_, log(ml/min/1.73^2^)	460,826	UKB	[27]	
** Urate**, mg/dL	457,690	CKDGen	[28]	Novel
** UACR**, log(mg/g)	564,257	UKB, CKDGen	[25]	
** Chronic kidney disease**	112,143/1,406,843	UKB, FG, MVP	[17]	Independent
**Lipid traits**				
** LDL-c**, mg/dL	1,494,170	UKB, GLCG	[29]	Updated
** HDL-c**, mg/dL	1,547,630			
** Triglycerides**, log(mg/dL)	1,521,780			
**Glycaemic traits**				
** Glucose**, mmol/L	394,976	UKB	[30]	Independent
** HbA1c**, mmol/mol	412,626			

*BCC* blood cell consortium, *BP* blood pressure, *CKDGen* chronic kidney disease genetics consortium, *eGFRcyst/crea* glomerular filtration rate estimated using cystatin-c/creatinine, *FG* FinnGen data version R12, *GIANT* Genetic investigation of anthropometric traits, *GLGC* global lipids genetics consortium, *HbA1c* glycated haemoglobin, *HDL-C* high-density lipoprotein cholesterol, *ICBP* international consortium of blood pressure, *LDL-C* low-density lipoprotein cholesterol, *MVP* million veteran program, *NPX* normalised protein expression units, *SUD* substance use disorder, *TE* total energy, *UACR* urine albumin-creatinine ratio, *UKB* UK Biobank. ^†^Comparison of data sources with those used in our prior investigation of FGF21. Novel refers to traits not investigated before. Blank cells indicate the use of similar datasets

### Selection of the genetic instrument

**Fig. 2:**
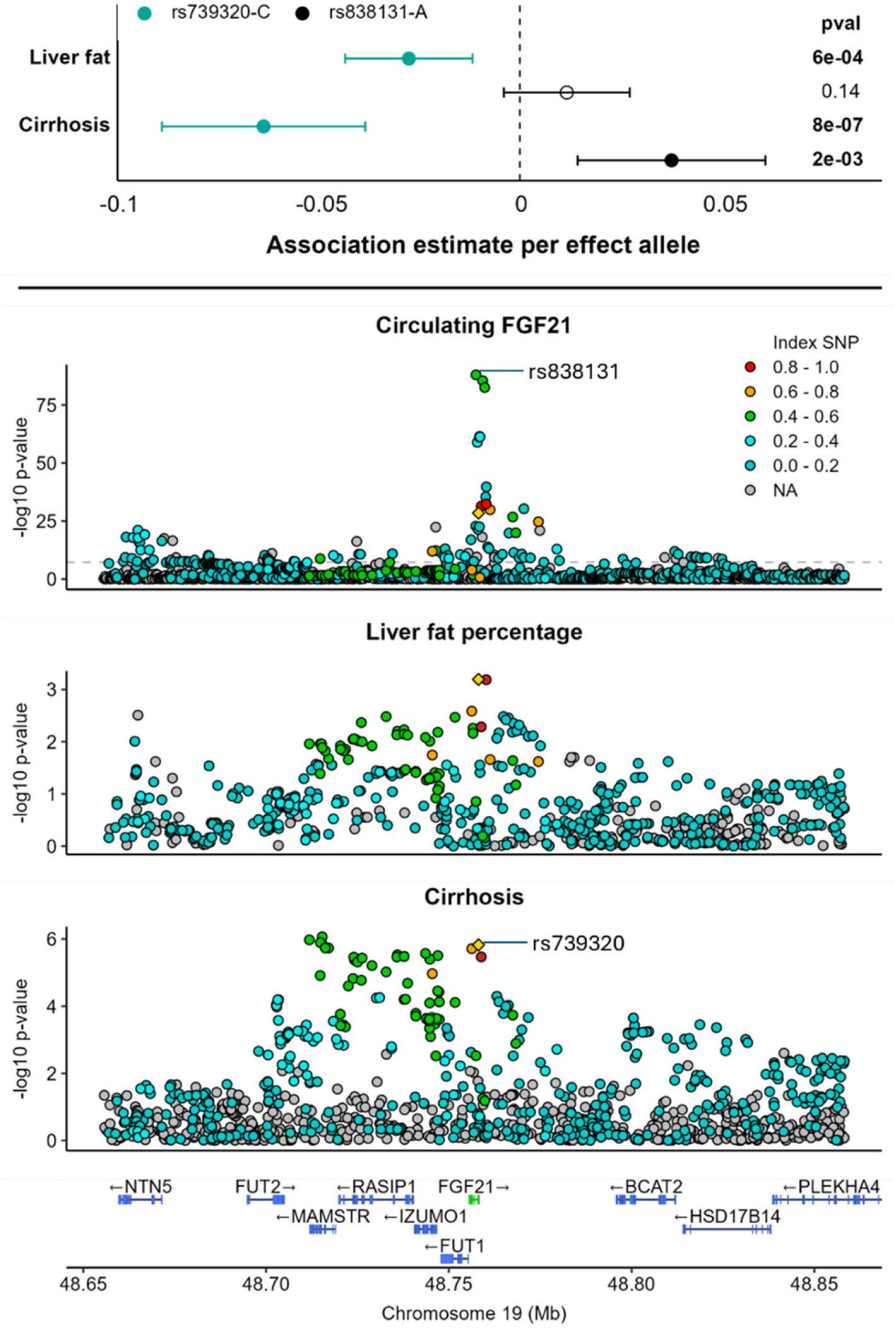
Genetic association evidence used to inform instrument selection. Top panel: Comparative genetic association estimates for rs838131-C and rs739320-C with liver fat percentage and cirrhosis risk. Bottom panel: Locus plot depicting associations between genetic variants in and around *FGF21* for circulating FGF21 levels, liver fat percentage and cirrhosis risk. The yellow triangle represents rs739320. Pairwise correlation coefficients (*r*^2^) with rs739320 are indicated by the colour of each data point

We considered two main candidate genetic instruments for FGF21 agonism. The first is an intronic cis-protein quantitative trait locus (cis-pQTL) variant that represents the strongest genetic association signal at the *FGF21* locus for circulating FGF21 levels (rs838131; 0.12 ± 0.006 higher normalised protein expression (NPX) units of FGF21 per A allele; *p* = 1 × 10^−88^).^13^ The other is the common allele *FGF21* L174P missense variant (rs739320) that represents the strongest genetic association signal at the *FGF21* locus for liver fat content (Fig. 2) and cirrhosis risk [11]. These variants are in weak linkage disequilibrium, with an *R*^*2*^ of approximately 0.5 in European cohort reference data [32].

Instrument validity was investigated by comparing associations of these two candidates across outcome traits known to be affected by treatment with FGF21 analogues in clinical trial settings, namely liver fat and liver cirrhosis [4]. Other potential instruments for FGF21 were also considered, and are discussed in Additional file 1: Supplementary Text.

### Genetic association estimates

Genetic association estimates are provided as betas and 95% confidence intervals (95% CI) per additional copy of the rs739320 C allele. The C allele is the major allele of rs739320 that is associated with lower liver fat percentage and cirrhosis risk.

Betas represent log-odds for binary outcomes, and standard deviation (SD) unit difference (95% CI) for continuous outcomes. Where original data sources reported unstandardised association estimates, adjustments were applied based on reported trait SDs (systolic blood pressure: 19 mmHg, diastolic blood pressure: 11 mmHg, pulse pressure: 13.7 mmHg, carbohydrate intake: 8.6%, fat intake: 6.9, protein intake: 3.5%, log-transformed cystatin C-based estimated glomerular filtration rate (eGFR): 0.2 ml/min/1.73^2^). Unadjusted *p*-values are presented throughout. For primary outcome traits, associations with *p*-values lower than 0.017 are considered statistically significant, after applying a Bonferroni correction to account for multiple testing of three outcomes.

### Colocalisation

We conducted colocalisation analyses to investigate whether the lead genetic association signal at the *FGF21* region (*FGF21*± 100 kb) for liver cirrhosis risk [12], is the same as that of other outcomes of interest. Aside from instrument selection, these analyses were only performed on outcomes that were statistically significantly associated with instrumented FGF21 agonism, to serve as an indicator of the probability that this evidence could be attributable to confounding through variants that are correlated to our genetic instrument due to linkage disequilibrium. Colocalisation analyses were performed on 369 variants that were represented in all considered datasets using the ‘Coloc’ package (v5.2.3) set to its default priors [33, 34]. This method does not incorporate information on linkage disequilibrium structure. The output comprises posterior probabilities (PP) for five competing hypotheses. The first three are that causal genetic predictors exist for neither (PPH0) or one of the two traits (PPH1 for trait 1 and PPH2 for trait 2). The third and fourth hypotheses are that causal genetic predictors exist for both traits and that these are distinct (PPH3) or overlapping (PPH4).

## Results

### Instrument identification

We observed that genetic association estimates considering FGF21 agonism instrumented through the lead pQTL, rs838131, contradicted clinical trial findings, as the plasma FGF21-increasing A allele did not significantly associate with liver fat percentage and was associated with higher risk of liver cirrhosis. In contrast, the C allele of rs739320 was strongly associated with both lower liver fat and lower cirrhosis risk (odds ratio = 0.94, 95% CI = 0.91 to 0.96) (Fig. 2).

Investigating the wider genomic region, *FGF21*± 100 kb, we confirmed that the genetic predictors of affinity-based measures of circulating FGF21 were not the same as those for liver cirrhosis liability (PPH3 = 0.88). It is therefore likely that FGF21 pQTLs may be confounded by the effects of neighbouring correlated variants in linkage disequilibrium, such as those affecting expression of the FUT2 gene [35].

Overall, considering that *FGF21*-rs739320 is a functionally annotated variant that mimics the effect of FGF21 analogues observed in clinical trials, all further analyses for the effects of FGF21 were performed using this variant.

Further consideration of genetic confounding through variants in linkage disequilibrium of rs739320 is provided in Additional file 1: Supplementary Text.

### Predicted effects of FGF21 agonism on alcohol consuming behaviours

**Fig. 3:**
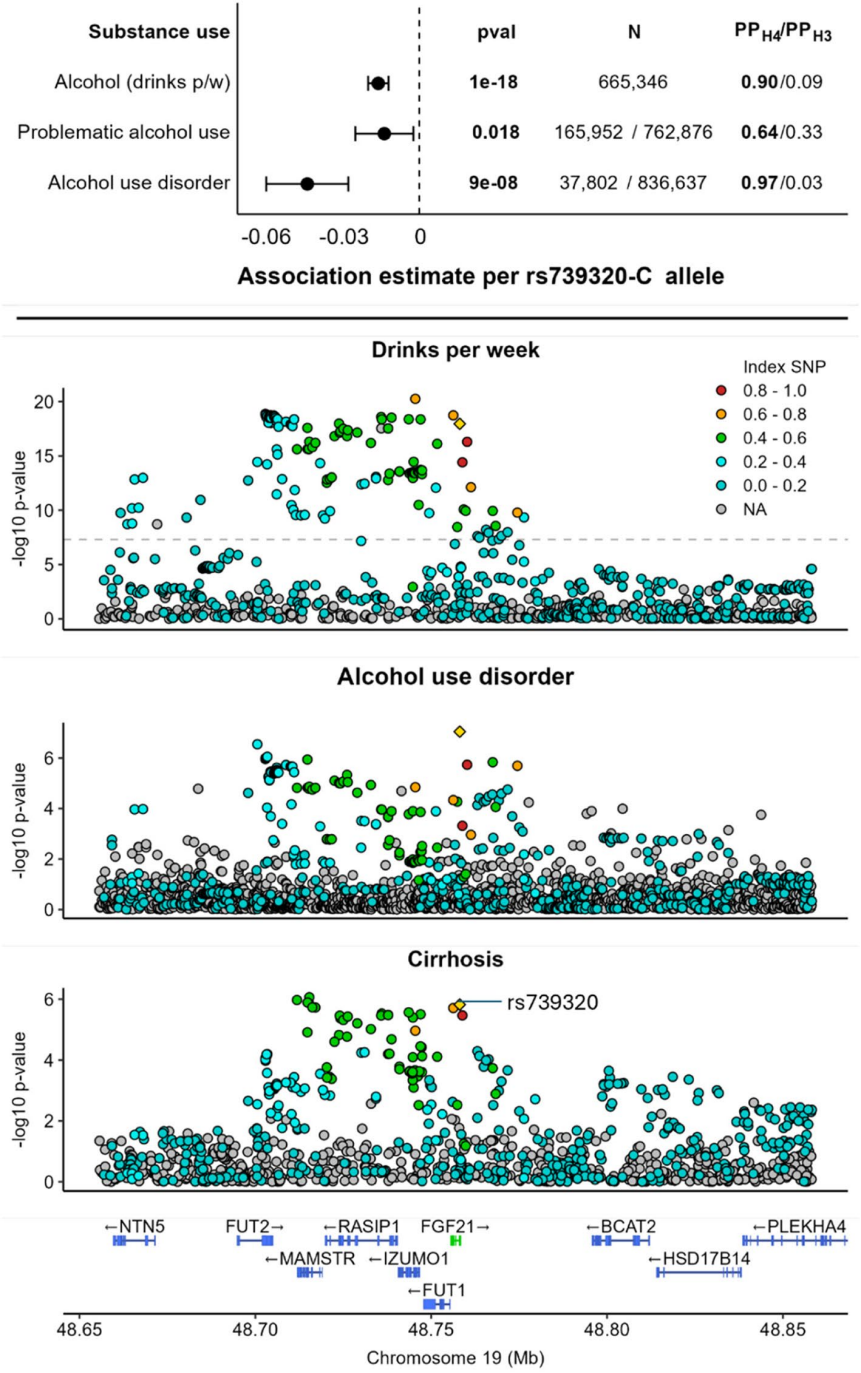
Genetic association evidence implicating FGF21 in alcohol consuming behaviours. Top panel: Genetic association estimates alongside unadjusted association *p*-values, sample size information and colocalization results. PPH4: Posterior probability that genetic predictors are shared with those for cirrhosis. PPH3: Posterior probability that genetic predictors are independent of those for cirrhosis. Bottom panel: Locus plot depicting associations between genetic variants in and around *FGF21* for alcohol consumption (drinks per week), risk of alcohol use disorder and cirrhosis risk. The yellow triangle represents rs739320. Pairwise correlation coefficients (*r*^2^) with rs739320 are indicated by the colour of each data point

We observed consistent associations between instrumented FGF21 perturbation and behavioural traits related to alcohol use. The C allele of rs739320, which is associated with lower liver fat percentage and cirrhosis risk, was also associated with lower alcohol consumption and lower risk of problematic alcohol use and AUD (Fig. 3). Colocalisation analyses confirmed the robustness of these findings. The potential effect of FGF21 agonism on addictive behavioural traits appears to be specific to alcohol consumption, as associations were not observed with cigarette smoking behaviour, risk of smoking dependency or risk of substance use disorder excluding alcohol (Additional file 1: Table S1).

### Predicted effects of FGF21 agonism on cardiometabolic risk factors

**Fig. 4:**
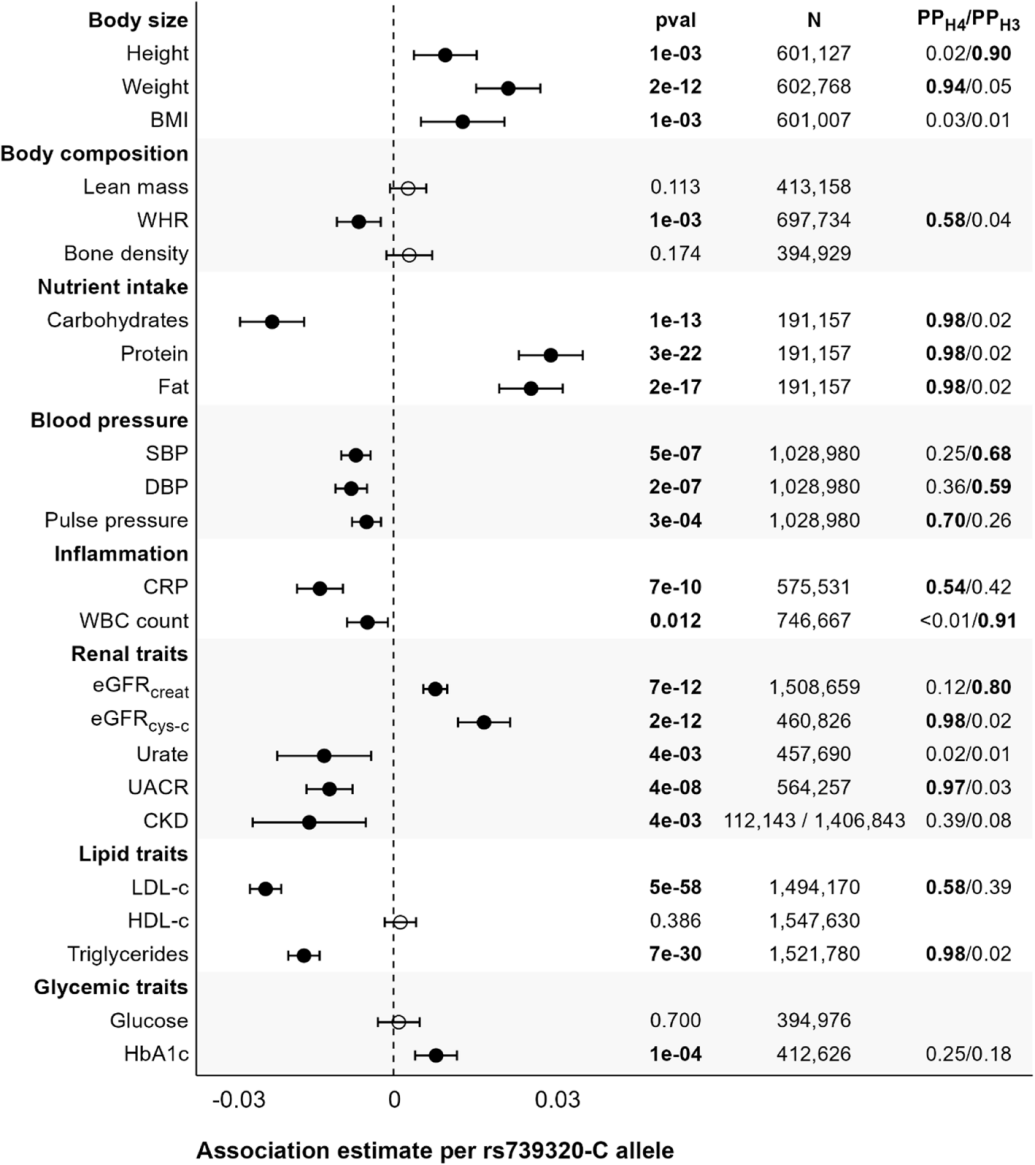
Genetic associations with biomarkers and risk factors of cardiometabolic and kidney disease risk. Association estimates represent change per additional rs739320 C allele as an instrument for FGF21 agonism. Association p-values, sample size information and colocalisation results are provided as well. PPH4: Posterior probability that genetic predictors are shared with those for cirrhosis. PPH3: Posterior probability that genetic predictors are independent of those for cirrhosis

Results for cardiometabolic and other traits are summarised in Fig. 4. Instrumented FGF21 agonism was associated with higher body size (height, weight and body mass index), but lower central adiposity as represented by waist-to-hip ratio (WHR). No association was observed with bone mineral density. Prior reported associations of genetically predicted FGF21 agonism with lower relative intake of carbohydrates and higher relative protein and fat intake were replicated [31]. Similarly, we replicated protective associations of instrumented FGF21 agonism with blood pressure traits, blood lipids, renal function, and chronic kidney disease risk (odds ratio: 0.98, 95% CI: 0.97 to 0.99; *p*= 0.004) [31], but observed a potential adverse association with glycated haemoglobin and no association with blood glucose. Finally, we observed lower white blood cell counts and C-reactive protein levels with genetically predicted FGF21 agonism.

Leveraging colocalisation analyses we report strong evidence to support that the genetic predictors of cirrhosis risk at the FGF21 locus overlap with those for body weight, WHR, macronutrient distribution, pulse pressure, C-reactive protein, cystatin C-based eGFR, urinary albumin-creatinine ratio and low-density lipoprotein cholesterol, and triglycerides. Data for CKD and serum urate and glycated haemoglobin did not have sufficient statistical power for colocalisation analyses.

## Discussion

### Main findings

In this study, we leveraged a missense variant in the *FGF21*gene that strongly associates with the known clinical effects of FGF21 analogues, namely lowering of circulating triglycerides, liver fat and risk of cirrhosis. We further show that while our chosen instrument does recapitulate the known clinical effects of FGF21 agonism, this is not observed with the lead cis-pQTL for FGF21. The discrepancy may be attributable to genetic confounding through effects of variants in linkage disequilibrium, such as those affecting expression of the neighbouring FUT2 gene [35].

Studying genetic associations, we provide consistent evidence to support favourable effects of FGF21 agonism on alcohol consumption behaviours, including alcoholic drinks per week, problematic alcohol use and AUD. Colocalisation analysis further supports that the findings for alcoholic drinks per week and AUD are unlikely to be due to confounding from variants in linkage disequilibrium.

Considering metabolic and dietary behaviours more generally, we provide human genetic and colocalisation evidence supporting effects of FGF21 on increasing growth (including both height and weight), improving body weight distribution (lowering WHR), lowering carbohydrate intake and increasing protein and fat intake, lowering C-reactive protein, improving kidney function (increasing eGFR and lowering urinary protein-creatinine ratio), and lowering LDL-C levels. We do not suggest that these findings are mediating any effect of FGF21 on lowering AUD risk, but rather that they may be representing the broad pleiotropic profile of FGF21. We find no genetic evidence supporting effects of FGF21 (favourable or unfavourable) on bone mineral density.

### Findings in context

AUD carries a significant societal burden, affecting approximately 30 million individuals globally and accounting for £249 billion (USD) annually in social and healthcare costs [36]. Few treatments have been approved for AUD [9], with the available medications having limited efficacy [37], and being underused [38]. While emerging evidence supports effects of glucagon-like peptide 1 analogues (GLP1) for reducing alcohol consumption [39], this is proposed to have a complementary pharmacological mechanism to FGF21 [6, 40], The diverse mechanism of action of FGF21 may offer considerable and additive benefit to currently available and exploratory mechanisms of action in AUD including approved opioid receptor antagonists and the emerging GLP-1 class [41].

Previous Mendelian randomisation analyses have supported favourable effects of FGF21 overlapping with those investigated in our current work [31]. However, this current study makes several notable advances. Firstly, we explore a greater number of traits related to alcohol intake behaviours, to increase conviction on the potential efficacy of FGF21 analogues for AUD. Secondly, studies with larger sample sizes are considered, to improve statistical power. Thirdly, we perform colocalisation analyses to help exclude the possibility of genetic confounding explaining our observed genetic association findings.

### Implications for further research

Clinical drug development has a failure rate of approximately 90%, largely due to the pre-clinical evidence typically being drawn from animal experiments and traditional epidemiological studies, which are limited in their human relevance and ability to draw causal inferences [42, 43]. Drug targets supported by human genetic data are more than twice as likely to advance to patient care [44], and such genetic insights are becoming increasingly accessible as the availability of large-scale genetic association datasets and genotyped biobanks grows. The findings of this study provide human genetic evidence supporting effects of FGF21 agonism on reducing alcohol consumption and risk of AUD, alongside a range of favourable metabolic effects, which complement the beneficial effects of FGF21 analogues on lowering triglycerides, liver fat and liver cirrhosis risk that have already been observed clinically [2, 4].

Our current findings also triangulate with the existing animal and observational data implicating FGF21 in regulating alcohol consumption as well as the effects of alcohol. Overexpression of FGF21 in transgenic mice results in lower alcohol preference [45], and pharmacological administration of FGF21 analogues reduces alcohol consumption in mice and non-human primate models [46]. There is also human epidemiological data linking FGF21 to AUD [47], although directionality of effect is difficult to infer without randomisation of an FGF21 intervention. Our current genetic findings strengthen the existing data by adding a causal framework in humans. Taken together, the totality of evidence strongly implicates FGF21 agonism as a promising therapeutic strategy for treating AUD.

### Strengths and limitations

Our work has several strengths. Firstly, the genetic associations can infer causality in humans, thus supporting the efficacy of FGF21 analogues for treating AUD in the clinic. Secondly, we complement the genetic association evidence with statistical colocalisation, to support that the findings are unlikely to be attributable to genetic confounding through neighbouring variants in linkage disequilibrium. Third, we consider a range of complementary traits related to alcohol consumption behaviours from distinct data sources, which all support similar conclusions to strengthen the body of genetic evidence. Finally, we leverage a plausible instrument, in contrast to the cis-pQTL for FGF21, which does not recapitulate known effects of FGF21 analogues when applied in the Mendelian randomisation framework.

Our work also has limitations. Firstly, the analyses were performed using data from largely European populations and so may not necessarily be extrapolated to other population groups. Secondly, the insights are limited by the available genetic association data. As such there may be the possibility of false negative findings for underpowered outcomes. Thirdly, the absence of colocalisation evidence may be attributable to low statistical power or confounding signals which may mask true shared causality, thus limiting interpretability. Fourthly, there remains the possibility of confounding through variants in linkage disequilibrium to our instrument variant explaining the observed associations (Additional file 1: Supplementary Text). Finally, these genetic analyses consider the effects of small lifelong variation in FGF21 agonism and may not reliably mimic the effect of a clinical intervention at a discrete timepoint in adult life. Caution should therefore be taken in extrapolating human genetic findings to the clinic.

## Conclusions

The study provides robust human genetic evidence supporting favourable effects of FGF21 agonism on risk of AUD and related outcomes. Further clinical study investigating FGF21 as a therapeutic target for this unmet clinical need and massive societal burden is duly warranted.

## Supplementary Information

Additional file 1. Table S1 and Supplementary Text. Table S1: Genetic associations with smoking behaviours and substance use risk not including alcohol. Supplementary Text: Fine-mapping of the FGF21 gene for cirrhosis risk. Consideration of alternative instruments for FGF21. Consideration of potential genetic confounding through variants in linkage disequilibrium. Consideration of rare variants. Discussion.

## Data Availability

This study used publicly available summary data that can be obtained from the cited original sources.

## Abbreviations

AUD: Alcohol use disorder
CI: Confidence interval
cis-pQTL: Cis-protein quantitative trait locus
eGFR: Estimated glomerular filtration rate
FGF21: Fibroblast growth factor 21
GLP1: Glucagon-like peptide 1
GWAS: Genome-wide association study
MASH: Metabolic dysfunction-associated steatohepatitis
NPX: Normalized protein expression
PAU: Problematic alcohol use
PP: Posterior probability
SD: Standard deviation
SNP: Single-nucleotide polymorphism
