# Supplementary material for: Robust human genetic evidence supporting causal effects of FGF21 on reducing alcohol consuming behaviours
Source: BMC Med. 2026 Mar 23;24:280. doi: 10.1186/s12916-026-04807-x (PMC13130737; doi:10.1186/s12916-026-04807-x)
Supplement: Supplementary file 1 — Additional file 1. Table S1 and Supplementary Text. Table S1: Genetic associations with smoking behaviours and substance use risk not including alcohol. Supplementary Text: Fine-mapping of the FGF21 gene for cirrhosis risk. Consideration of alternative instruments for FGF21. Consideration of potential genetic confounding through variants in linkage disequilibrium. Consideration of rare variants. Discussion. [file 12916_2026_4807_MOESM1_ESM.docx]

**Robust human genetic evidence supporting causal effects of FGF21 on reducing alcohol consuming behaviours**

Supplementary Materials

**Table S1.** Genetic associations with smoking behaviours and substance use risk not including alcohol.

| Outcome | Beta | SE | p-value |
| --- | --- | --- | --- |
| Smoking, cigarettes per day | 0.004 | 0.003 | 0.152 |
| Smoking dependency, log-odds | -0.005 | 0.010 | 0.584 |
| SUD excl. alcohol, log-odds | -0.009 | 0.017 | 0.609 |

SUD: substance use disorder. Association estimates represent change per additional rs739320 C allele as an instrument for FGF21 agonism

**Supplementary Text**

***Fine-mapping of the FGF21 gene for cirrhosis risk***

We performed fine-mapping of the *FGF21* gene using the Sum of Single Effects (SuSiE) regression framework. We generated the reference panel from 490,541 UK Biobank participants with Whole Genome Sequencing data and used information of inferred genetic ancestry (Hu.S. Nature Genetics 2025) to randomly select 10,000 participants with more than 90% European ancestry. Summary level genetic association data for cirrhosis risk at the FGF21 gene were obtained from Ghouse J, et al., Nature Genetics 2024, which included 1,580,011 (15,225 cases/ 1,564,786 controls). A total of 759 variants were available considering 50kb around the FGF21 gene. Unfortunately, this fine-mapping analysis did not identify any credible set at the FGF21 gene for cirrhosis risk.

***Consideration of alternative instruments for FGF21***

In the Open Targets Platform, other cis-pQTL or liver cis-eQTL variants are reported from independent, fine-mapped credible sets, such as: rs2231861 (5’UTR FGF21, cis-pQTL) and rs2548957 (upstream FUT1, but downstream of FGF21 in forward strand; liver cis-eQTL). However, these do not associate with liver fat, and as FGF21 analogues have been shown to lower liver fat in clinical trials, we do not believe that they can serve as valid instruments for the effects of FGF21. They also have weaker associations than the L174P variant with cirrhosis risk, which is another positive control outcome.

| **Trait** | **SNP** | **chr** | **pos** | **Effect Allele** | **Other Allele** | **Effect Allele Frequency** | **Beta** | **Standard Error** | **P-value** |
| --- | --- | --- | --- | --- | --- | --- | --- | --- | --- |
| Cirrhosis | rs2231861 | 19 | 48756064 | G | C | 0.06 | -0.078 | 0.026 | 2.37E-03 |
| Cirrhosis | rs2548957 | 19 | 48759243 | G | A | 0.53 | 0.024 | 0.013 | 5.65E-02 |
| Cirrhosis | rs739320 | 19 | 48758111 | C | T | 0.64 | -0.063 | 0.013 | 1.50E-06 |
| Cirrhosis | rs838131 | 19 | 48757420 | C | A | 0.50 | -0.038 | 0.013 | 2.99E-03 |
| Liver fat | rs2231861 | 19 | 48756064 | C | G | 0.94 | 0.019 | 0.016 | 2.50E-01 |
| Liver fat | rs2548957 | 19 | 48759243 | G | A | 0.57 | 0.004 | 0.008 | 6.40E-01 |
| Liver fat | rs739320 | 19 | 48758111 | T | C | 0.40 | 0.028 | 0.008 | 6.40E-04 |
| Liver fat | rs838131 | 19 | 48757420 | A | C | 0.54 | 0.012 | 0.008 | 1.40E-01 |

The same data sources are used as in the main manuscript.

***Consideration of potential genetic confounding through variants in linkage disequilibrium***

We also explore potential confounding through variants in linkage disequilibrium (LD) explaining the observed associations for L174P. Using the LDlink tool ([LDlink | LDproxy](https://ldlink.nih.gov/ldproxy?ref=25403)), we identify two SNPs in linkage disequilibrium r^2^>0.8 with L174P in European ancestry individuals: rs9917102 (r^2^=0.88) and rs2081194 (r^2^=0.85). Searching The Open Targets Platform database, rs9917102 is in the molecular quantitative trait locus (QTL) credible set for only *FAM83E* gene expression in the dorsolateral prefrontal cortex (PMID 28869584), and rs2081194 is not in the credible set for molecular QTLs for any gene. Colocalization analyses do support that *FAM83E* gene expression in the dorsolateral prefrontal cortex may have a shared causal mechanism with alcohol consumption (PMID 3064325, colocalization posterior probability H4 = 89%). This therefore highlights the potential for genetic confounding through FAM83E explaining the effect of alcohol consumption and related traits, rather than FGF21.

In contrast, no SNPs are identified to be in LD r^2^>0.8 with L174P in African ancestry populations, reducing the potential for genetic confounding through FAM83E. Associations of rs739320 C allele with alcohol consumption in African ancestry individuals as compared to European ancestry individuals (PMID 36477530) are detailed below:

| **SNP** | **Effect allele** | **Allele frequency in 1000G** | **Beta** | **Standard Error** | **P** | **N** | **Ancestry** |
| --- | --- | --- | --- | --- | --- | --- | --- |
| rs739320 | C | 0.956127 | -0.0289 | 0.023 | 0.21 | 8078 | AFR |
| rs739320 | C | 0.633201 | -0.0158 | 0.002 | 1.11E-18 | 665346 | EUR |

While the estimate is of greater magnitude, with less precision likely due to the smaller sample size and less statistical power, it does not reach statistical significance in the African ancestry population.

***Consideration of rare variants***

As rare variants may be less susceptible to genetic confounding, *FGF21* rare variant associations with alcohol intake were also studied. To investigate this, we interrogated Genebass, which is a resource that compiles exome-based association statistics obtained from 394,841 individuals with available data from the UK Biobank (<https://app.genebass.org/about>). Results are presented below for the outcome of alcohol intake, which was calculated by summing the total intake for grams of alcohol through red wine intake, white wine intake, beer intake, fortified wine intake, spirits intake and other alcoholic drinks intake. Results for the ten variants with strongest statistical evidence of an association are presented below.

| **Variant ID** | **Consequence** | **Aimon Acid Change** | **Raw P-value** | **Allele Count** | **Homozygote Count** | **Allele Frequency** |
| --- | --- | --- | --- | --- | --- | --- |
| 19-48758111-T-C | missense | Leu174Pro | 6.26E-09 | 446122 | 135402 | 6.06E-01 |
| 19-48756272-A-G | synonymous | Gly12= | 1.02E-08 | 405336 | 111930 | 5.51E-01 |
| 19-48757028-C-T | missense | Ser113Leu | 0.003 | 9 | 0 | 1.22E-05 |
| 19-48756442-T-C | missense | Val69Ala | 0.010 | 1 | 0 | 1.36E-06 |
| 19-48756376-G-A | missense | Arg47Gln | 0.014 | 10 | 0 | 1.36E-05 |
| 19-48756968-T-C | missense | Ile93Thr | 0.015 | 1 | 0 | 1.36E-06 |
| 19-48756946-T-C | synonymous | Leu86= | 0.016 | 7 | 0 | 9.51E-06 |
| 19-48758116-G-C | missense | Glu176Gln | 0.020 | 1 | 0 | 1.36E-06 |
| 19-48756271-G-A | missense | Gly12Glu | 0.027 | 1 | 0 | 1.36E-06 |
| 19-48758145-C-T | synonymous | Pro185= | 0.030 | 1 | 0 | 1.36E-06 |

The L174P common variant that we used as an instrument shows an association with alcohol intake after false-discovery rate correction for 229 variants tested, and the common variant with the second strongest association is in linkage disequilibrium (r^2^ = 0.635). This search did not identify rare variants that implicate FGF21 in affecting alcohol intake.

***Discussion***

We have made various attempts to exclude confounding from variants in linkage disequilibrium as an explanation for the association between our selected instrument for FGF21, missense variant L174P, and alcohol intake behaviours. However, limitations remain:

- Locus plots at *FGF21* for the liver cirrhosis positive control trait shows plateau-like association signals, and as standard coloc methods do not explicitly model linkage disequilibrium structure, it is difficult to exclude the possibility that colocalization is driven by multiple correlated variants rather than L174P specifically.
- Fine-mapping was performed, but did not meaningfully resolve the linkage disequilibrium structure in the region.
- Genetic associations were examined in the largest and most recent meta-analyses, where L174P remains the lead variant; however, it is unknown whether the plateau-like association pattern persists as summary GWAS data are not available.
- Exploration of alternative candidate genes identified a potential confounder (FAM83E), which raises the possibility of confounding.
- Analyses in non-European populations were attempted, but low allele frequencies and limited sample sizes rendered these results inconclusive.
- Rare variants implicating FGF21 in alcohol intake were not identified in UK Biobank.

Thus, while this genetic evidence alone cannot completely exclude the possibility of confounding affecting the association of L174P with alcohol intake traits, the findings should be interpreted in the context of the wider body of biological evidence, which do support a protective effect of FGF21.
